# Supplementary material for: Gamble for the needy! Does identifiability enhances donation?
Source: PLoS One. 2020 Jun 30;15(6):e0234336. doi: 10.1371/journal.pone.0234336 (PMC7326157; doi:10.1371/journal.pone.0234336)
Supplement: S1 Appendix — (PDF) [file pone.0234336.s001.pdf]

S1 Appendix : Stimuli and Apparatus in detail

Stimuli

The need game was embedded in a lottery with probabilities $p = .3$, .4, .6, and .7, of winning a bet and framed either as gain or as loss. In a gain frame, the probability p of winning the lottery appeared as green area in a pie chart; the probability of losing it, $1 - p$, was coded as the remaining gray area (Fig 1, left panel). In a loss frame, the probability of winning the lottery p appeared as gray area, and the probability of losing it, $1 - p$, was marked in red (Fig 1, right panel). Note that, both lotteries are identical; only the color coding is different. Each probability was presented 25 times in each frame, amounting to a total of 200 trials.

Below the charts are shown different amounts from which the donor can select one to bet on. In the current experiment three amounts (points) were offered: $x_1 = 1$, $x_2 = 10$, and $x_3 = 50$. L, C, and R in front of the amounts refer to the keys on a response box (left, center, right, see Apparatus) to indicate the donor's choice for one given trial.

The third row of the display in Fig 1 shows the current endowment and the need level of the donor (left hand side and labeled as "you") and of the recipient (right hand side and labeled as "Other person"). In this example, the donor's current endowment is 2,350 points (actually, the initial endowment) and the one of the recipient is 0; the need level for both players is 2,200 points. The need level of 2,200 points was determined according to the following rationale. In each of the 200 lotteries, the average amount to bet on was $(1 + 10 + 50)/3 = 20.33$ points. Accordingly, the expected outcomes of lotteries with probabilities .3, .4, .6, and .7 are 6.099, 8.132, 12.198, and 14.231, respectively. For calculating the need level, the expected outcomes were rounded up to 7, 9, 13, and 15, respectively. The rounding-up was done to create a need level that is a bit above the expected value and could not be reached incidentally. The need level (2,200) is then the sum of the rounded up expected outcomes of 200 lotteries. The donor's endowment of 2,350 points was 150 points above the need threshold. The exact value is arbitrary but with the following consideration: even if the donor chooses always the lowest amount of 1 point and, in the worst case, would lose each of the 200 rounds, the need threshold would be unattainable.

Note that, during the experiment, the displayed endowment changes for both players after each round whereas the need display remains constant during one block of trials.

At the bottom of the display is a white bar changing its size as a function of the remaining time on a given trial.

The recipient, labeled as "Other person", had three levels of identifiability. The recipients were either referred to as "Other person" only without further description or as a person described on a paper-written profile with information about name, age, place of residence, and occupation (paper size 176mm × 250mm, ISO standard B5). The profile additionally contained a picture. Specifically, the person described was a 68 years old pensioner from Bremen Gröpelingen. His first name was "Olaf". The description is based on a real person who was also present in some conditions described in detail below. The three conditions are referred to as "no-ID, picture-ID", and "person-ID" .

Apparatus

Stimulus presentation and response registration were controlled by one of six computer systems. Two of them were running a 64bit Linux OS (Linux Mint v. 17 and Linux

Ubuntu v. 12.10) with a standard 22″ LCD wide-screen monitor (screen resolution: 1920×1080 pixels). The other four computer systems were running a Windows 7 64bit OS, two with the same monitor type as the Linux system and two with a smaller standard 17″ LCD monitor (screen resolution: 1280×960 pixels). The control software operated on Matlab® 2014a and 2017 including the Psychophysics Toolbox version 3.0.13. The input device was a USB 2.0 button box with three buttons (L: left, C: Center, R: Right). In a few exceptional cases a standard keyboard was used, where the participants made their choice by pressing the "Y" (on a German keyboard), "V" or "M" keys for L, C, and R, respectively.

The monitor display had a black background. The instruction text was written in white. Text font was "Helvetica". In the center of the screen, a light blue rectangular window was displayed with the size of 800 × 700 pixels. Within this window, the lotteries were represented in pie charts with a green colored proportion that indicates the winning probability in gain frames and a red colored proportion that indicates the loss probabilities in loss trials. The diameter of the pie charts was 300 pixels. The choice options (three different stakes) were presented in black in a font size of 34pt below the pie charts. Endowment and need of the participant and the passive individual were displayed under the choice options on the left and right side of the rectangular window, respectively. Available time for making a decision was indicated by a white, horizontal status bar which decreased continuously from 400 pixels to 0 pixels within the given time limit. The bar was displayed below the light blue rectangular window (see Fig 1 for screenshots of the display).
