# Supplementary material for: Gamble for the needy! Does identifiability enhances donation?
Source: PLoS One. 2020 Jun 30;15(6):e0234336. doi: 10.1371/journal.pone.0234336 (PMC7326157; doi:10.1371/journal.pone.0234336)
Supplement: S2 Appendix — (PDF) [file pone.0234336.s002.pdf]

S2 Appendix : Descriptives of Experiment 1 in detail

Table A1 shows the descriptive analysis of how often the three different options, i.e. bets, were chosen given the experimental conditions across experimental groups (between-subject conditions). The pattern is consistent across all four experimental groups and among the conditional frequencies: The small amount (1 point) was the most frequently chosen option and the medium amount (10 points) the least chosen one.

Table A1. Experiment 1: Relative choice frequencies for between-subject conditions and conditional frequencies of conditions Identity among Need.

	Relative choice frequencies		
Between-subject conditon	1 point	10 points	50 points
Need (0)	.43	.23	.33
Need (2,200)	.47	.21	.32
Identity (no-ID)	.47	.23	.31
Identity (picture-ID)	.43	.21	.35
no-ID/Need (0)	.47	.23	.30
picture-ID/Need (0)	.41	.23	.36
no-ID/Need (2200)	.47	.23	.31
picture-ID/Need (2200)	.46	.19	.35

Table A2 shows the descriptive statistics for the within-subject conditions Frame, Probability and Time. The frequency to bet on larger amounts decreases for winning probabilities smaller than 0.5 and increase for winning probability larger than 0.5. For the remaining factors (Frame and Time) the small amount was the most frequently chosen option.

Table A2. Experiment 1: Relative frequencies for within-subject conditions.

	Relative choice frequencies		
Within-Subject condition	1 point	10 points	50 points
Gain (Frame)	.38	.24	.38
Loss (Frame)	.52	.21	.27
.3 (Probability)	.78	.13	.09
.4 (Probability)	.64	.24	.12
.6 (Probability)	.23	.35	.42
.7 (Probability)	.15	.17	.68
1s (Time)	.47	.20	.33
3s (Time)	.44	.24	.32

Table A3. Experiment 1: Relative frequencies for conditional frequencies.

	Relative choice frequencies		
Conditional frequencies	1 point	10 points	50 points
Gain/1s	.39	.22	.39
Gain/3s	.37	.25	.38
Loss/1s	.54	.19	.27
Loss/3s	.50	.22	.27
Gain/0-Need	.36	.25	.39
Gain/2,200-Need	.40	.22	.37
Loss/0-Need	.51	.21	.27
Loss/2,200-Need	.53	.20	.27
Gain/no-ID	.39	.25	.37
Gain/picture-ID	.37	.22	.40
Loss/no-ID	.55	.21	.25
Loss/picture-ID	.49	.20	.30

Moreover, among the conditional frequencies (shown in Table A3) the same pattern can be observed. However, for very few conditions (picture-ID condition, Need 0, time limit 3s, all under a gain frame) the smallest amount was chosen most frequently.
