# Supplementary material for: Gamble for the needy! Does identifiability enhances donation?
Source: PLoS One. 2020 Jun 30;15(6):e0234336. doi: 10.1371/journal.pone.0234336 (PMC7326157; doi:10.1371/journal.pone.0234336)
Supplement: S3 Appendix — (PDF) [file pone.0234336.s003.pdf]

S3 Appendix: Descriptives of Experiment 2 in detail

Overall, participants chose the large amount (50 points) most often and the medium amount (10 points) least often. Furthermore, participants chose the large amount most often in gain frames and the small amount most often in loss frames. Choice frequencies across probabilities were similar to those found in Experiment 1. The choice behavior in both Time conditions was similar to the overall pattern and it was stable for gain and loss frames among the different time limits. The relative choice frequencies for within-subject conditions and conditional frequencies can be found in Table A4.

Table A4. Experiment 2: Relative frequencies for within-subject conditions and conditional frequencies.

Condition	1 point	10 points	50 points
Gain (Frame)	.29	.25	.46
Loss (Frame)	.42	.24	.34
.3 (Probability)	.59	.23	.18
.4 (Probability)	.49	.30	.22
.6 (Probability)	.19	.30	.51
.7 (Probability)	.14	.16	.71
1s (Time)	.36	.23	.41
3s (Time)	.35	.26	.39
Gain/1s	.29	.23	.48
Gain/3s	.29	.26	.45
Loss/1s	.42	.23	.35
Loss/3s	.41	.26	.33
