# Supplementary material for: Gamble for the needy! Does identifiability enhances donation?
Source: PLoS One. 2020 Jun 30;15(6):e0234336. doi: 10.1371/journal.pone.0234336 (PMC7326157; doi:10.1371/journal.pone.0234336)
Supplement: S4 Appendix — (PDF) [file pone.0234336.s004.pdf]

S4 Appendix: Combined analysis (mixed-effects models) of Experiment 1 and 2 in detail

Data of 97 participants are included in the analysis. Data of 3.5 sessions (7 blocks) were lost due to computer failure. 345 trials were timeouts, leaving a total of 75,855 observations (including 45,620 observations from Experiment 1 and 30,235 from Experiment 2). The mixed-effects model analysis included Need, Identity, Frame, Probability and Time as fixed factors and participant and experimental block as random factors.

The analysis revealed that dictators pleaced significant higher bets in condition person-ID as compared to conditions no-ID and picture-ID. Moreover, we found the same effects of Framing and Probabilities as described in Experiment 1 and 2. No effect of Time was found. The interaction analysis revealed no significant interaction effects. The results of the mixed-effects model analysis is shown in Table A4. Model 1 determines main effects and Model 2 considers 2-way interaction effects.

Table A4. Combined analysis: Cumulative link mixed-effects Model 1 and Model 2.

	Model 1				Model 2			
Factor:	Est.	SE	z-value	p-value	Est.	SE	z-value	p-value
Identity (picture-ID)	.088	.176	.502	.616	.114	.176	.649	.517
Identity (person-ID)	.401	.151	2.656	.008	.393	.151	2.598	.009
Frame (gain)	.461	.009	49.352	$<.001$	.466	.018	26.194	$<.001$
Probability (.4)	.291	.013	21.822	$<.001$	.291	.013	21.826	$<.001$
Probability (.6)	1.393	.013	103.849	$<.001$	1.393	.013	103.848	$<.001$
Probability (.7)	1.947	.014	136.516	$<.001$	1.947	.014	136.518	$<.001$
Time (3s)	.032	.020	1.579	.114	.035	.022	1.573	.116
Frame×ID (picture-ID)					−.044	.025	−1.796	.073
Frame×ID (person-ID)					.017	.021	.827	.408
Frame×Time					−.006	.018	−.299	.765
Intercept 1 (1|10)	1.037	.109	9.532		1.042	.109	9.556	
Intercept 2 (10|50)	1.896	.109	17.413		1.901	.109	17.420	

Number of observations: 75855. Groups (random effects): Participants, 97; Blocks, 4. Dependent variable: choice frequencies. Reference categories of independent variables: Identity (recipient: no-ID), Frame (loss), Time (1s), and Probability (.3). Intercept 1 and 2 are threshold coefficients (cut points).
