# Supplementary material for: Gamble for the needy! Does identifiability enhances donation?
Source: PLoS One. 2020 Jun 30;15(6):e0234336. doi: 10.1371/journal.pone.0234336 (PMC7326157; doi:10.1371/journal.pone.0234336)
Supplement: Attachment — Submitted filename: Response to Reviewers.docx [file pone.0234336.s005.docx]

Dear Dr. Capraro,

We are very grateful for an extremely constructive and thoughtful set of reviews and the opportunity to resubmit. We append a detailed record of all the changes below.

Thank you

Marc Wyszynski on behalf of all authors

**Response to Editor and Reviewer**

**(Replies are in red and start with *)**

Editor comments:

1) Discussion about framing effects in the DG. I've done myself quite a lot of work on this topic recently, showing that moral frames dramatically impact DG behaviour (Capraro & Vanzo, 2019; Capraro et al. 2019; Bilancini et al. 2020). Of course, citing these works is not a requirement, but I'm mentioning them because they seem very related to your discussion.

*Thank you very much for referring to your interesting work. We took it up in the discussion pointing to future directions.

2) Discussion about the effect of time pressure on DG behaviour. Sorry to say this, but this part is largely incomplete. I happen to know this research very well (see my review: Capraro, 2019). The paper Cappelen et al. is a correlational paper and not a causal paper. The review by Evans et al also look at the correlations plus it is about cooperation in the public goods game and not altruism in the dictator game. Your setting is different: time pressure and DG. You correctly acknowledge that imposing a time limit and looking at response times are different thing, but you did not mention at all the huge literature on time pressure and DG. In this setting, there are two meta-analyses (Rand et al. 2016; Fromell et al 2020) and a review (Capraro, 2019), plus many individual papers.

*We agree that our setting is different. With respect to time, time pressure, and dual processes we would not agree with Rand et al. (see also the criticism by Ian Krajbich and an article on modeling time and dual processes (Diederich & Trueblood, Psych Rev, 2018, Vol. 125, No. 2, 270–292)). For not confusing the reader we dropped the entire part on reaction time. Nevertheless, your review article appears relevant.

**Reviewer #1**:

Overall, I thought this was an interesting article with a new game that has the potential to study new and interesting behavioral questions. The methods and analyses are strong. I believe this will be a valuable addition to the literature, once some concerns are addressed.

*Thank you very much for the kind remarks.

The payment structure of the game is unclear. The way I am understanding it is that if the donor meets their minimum need they are paid, regardless if the recipient’s need is met? Likewise, the recipient is paid if their need is met, irrespective of if the need is met of the donor?

*This is correct. We are sorry for any confusion. We explicitly state this now in the description of the rules.

The methods are unclear. There are too many details in the main text that are not necessary to understand the study, such as the apparatus and much of the text under stimuli. Simplify to make the main text as clear as possible, and relegate all additional details to a supplement. This would greatly improve the readability of the manuscript.

*Thank you very much for the suggestions. We moved the detailed descriptions to the supplement.

I am concerned about the framing of this game as similar to charity-lotteries. To begin, participants did not have a chance to not gamble and walk away, like they do in charity-lotteries. This constrains the choices of when and why people will choose to risk. Additionally, the recipient was an individual, not a charity. Moreover, the need of the recipient is largely unknown to people participating in charity lotteries, and they are not the sole person responsible for the outcome of the recipient. Lastly, the lottery would still receive the money spent, even if their ‘need’ is not met. These points should be discussed to clarify how the comparison between charity-lotteries and the need game can be made.

*This is all true. We used the charity lottery mainly as a motivational example. We point to these issues when describing the need game.

This paper does not incorporate much theory or prior work on relevant topics, such as the literature on needs-based helping and risk-taking, which address when and why people will take risks. I’ve offered some references below to help with this. To be clear, I do not require the authors to cite these specific papers, rather I am trying to point them in relevant directions to strengthen the paper. Authors could include the suggested papers if they feel it would strengthen their arguments.

*We wanted to keep the focus on a few distinct features. True, we could always expand it to a broader scope. Thank you for referring to the work by Mishra and colleagues that seems highly relevant.

The paper would benefit from a paragraph or two describing how this game could address new and interesting research questions beyond the ones presented in the current paper.

*This is now added at the end of the discussion.

The title of the article (or abstract) is misleading. What is the main focus of the paper? The title suggests that identifiability is the main focus, but identifiability is not even mentioned in the abstract. Likewise, the abstract is solely focused on the game, and none of the tested predictions in the paper. These both need to be re-framed to focus on the main points of the paper.

*We changed the title and the abstract accordingly.

The fact that if the minimum need is not met both players receive nothing needs to be noted earlier, including in the abstract. This point was unclear, and should be noted earlier to really understand the game.

*Again, sorry not being precise enough. We clarify this in the description of the need game.

What do you mean by ‘relative deprivation’ on p.2, line 35? This term is used differently by different groups of researchers.

*We changed it to avoid any confusion.
