# Supplementary material for: Gamble for the needy! Does identifiability enhances donation?
Source: PLoS One. 2020 Jun 30;15(6):e0234336. doi: 10.1371/journal.pone.0234336 (PMC7326157; doi:10.1371/journal.pone.0234336)
Supplement: Attachment — Submitted filename: Response_to_Editor_rev2.docx [file pone.0234336.s006.docx]

Dear Dr. Capraro,

Thank you again for handling our article and for the opportunity to make further changes that will surely improve the article. We append a detailed record of all the changes below.

Sincerely,

Marc Wyszynski on behalf of all authors

**Response to Reviewer**

**(Replies are in red and start with *)**

**Reviewer #1**:

I am pleased to see the vast improvements in the manuscript, which has resulted in a much clearer paper. I still have a few minor comments, which I hope help improve the paper further.

*Thanks again for your kind and constructive remarks.

1. When you create parallels between the need-game and charity lotteries, the connection needs to be made clear for the reader. Why is this game appropriate for studying charity-lotteries? As I pointed out in my last review, there are many differences, so highlight the similarities when you introduce these ideas (abstract, intro), otherwise it is difficult to draw the connection. In particular, the paragraph starting at line 23, p.2 starts with charities lotteries, then discusses a specific recipient. There seems to be a disconnect.

*Thank you for this suggestion. We modified the respective paragraph to make the connection clearer.

2. The description of the interaction effects in the results section is not sufficient to fully understand what is going on. For example, do you mean that for the 2200-need condition in experiment 1, people still made higher bets in gain frames than on the 0-need condition? Perhaps a figure would make this easier to understand.

*Yes, the framing effect is observable across both need conditions. It was stronger without a need. We added a further note which should better describe this effect.

3. I would recommend putting all appendices into a single document for ease of access and reading.

*Thank you for the recommendation. After careful consideration, we decided not to combine the appendices because separate documents make the reference to the respective appendix clearer when reading the body text (e.g. through the hyperref-function, which is activated in the PlosOne-Latex template). Furthermore, the content of the appendices is clearly listed in the "supporting information" section. We believe, therefore, that a single document would not improve accessibility and clearness.
